# Thirty-Day Thermomechanical Ageing of 3D-Printed and Thermoformed Orthodontic Aligners: A Comparative Force Analysis

**DOI:** 10.1016/j.identj.2026.111093

**Published:** 2026-09-01

**Authors:** Tarek Elshazly, Abdulaziz Alhotan, Ammar Al Shalabi, Ahmad Elshennawy, Hala Diraz, Kathrin Puchert, Hoon Kim, Sameh Talaat, Sinan Şen

**Affiliations:** a Department of Orthodontics, University Hospital Schleswig-Holstein, Kiel, Germany; b Oral Technology, Dental School, University Hospital Bonn, Bonn, Germany; c Department of Dental Health, College of Applied Medical Sciences, King Saud University, Riyadh, Saudi Arabia; d Department of Orthodontics and Pediatric Dentistry, Hamdan Bin Mohammed College of Dental Medicine (HBMCDM), Mohammed Bin Rashid University of Medicine and Health Sciences (MBRU), Dubai, UAE; e Innovative Digital Center for Orthodontics, The British University in Egypt, Al Shorouk City, Egypt; f Department of Orthodontics, Faculty of Dentistry, Ain Shams University, Cairo, Egypt; g Research Institute of Agriculture and Life Sciences, College of Agriculture and Life Sciences, Seoul National University, Seoul, Republic of Korea; h Department of Orthodontics, Future University, Cairo, Egypt

**Keywords:** Biomechanics, Orthodontic force, Removable orthodontic appliances, Materials testing, Dental materials

## Abstract

**Objectives:**

To investigate the effect of thermomechanical ageing on the force system of three-dimensional (3D)-printed orthodontic aligners compared with conventional thermoformed aligners and to evaluate the thickness characteristics of both aligner types.

**Materials and methods:**

Ten aligners were fabricated from 3D-printed Tera Harz TC-85 resin, and 10 from thermoformed Zendura FLX (ZF). All aligners underwent thermomechanical ageing for up to 30 days. Forces transmitted to the maxillary right central incisor (Tooth 11) in a resin model were measured before ageing (Day 0) and after thermomechanical ageing (Days 2, 7, and 30) using Fuji pressure-sensitive films to assess contact normal forces and an orthodontic measurement and simulation system (OMSS) to measure resultant 3D forces. Aligner thickness was evaluated using a digital caliper and microcomputed tomography.

**Results:**

TC-85 aligners generated significantly higher initial normal contact forces than ZF aligners (149.7 ± 25.6 N vs 69.8 ± 9.0 N). Both materials exhibited significant force decay following thermomechanical ageing. Orthodontic measurement and simulation system analysis demonstrated comparable preageing resultant forces for both materials (0.1 ± 0.07 N to 0.5 ± 0.09 N), with no statistically significant difference in facial force components at baseline. Facial forces remained relatively stable over time, whereas lingual resultant forces decreased significantly. Thickness analysis demonstrated pronounced thinning in ZF aligners (≈0.5 ± 0.05 mm) and thickening in TC-85 aligners (≈1.0 ± 0.09 mm).

**Conclusions:**

TC-85 aligners produced higher initial normal contact forces than ZF aligners, consistent with their greater postmanufacturing thickness and reported improved adaptation to the dental model; however, both materials exhibited force decay over time. Although preageing resultant forces were comparable between materials, the reduction in lingual resultant forces may adversely affect the predictability of tooth movement during prolonged aligner wear.

**Clinical significance:**

The current findings underscore the significant impact of aligner material, thickness, and manufacturing method on force stability and consistency in clear aligner therapy.

## Introduction

The increasing use of clear aligner therapy in orthodontic treatment has encouraged a growing wave of evolution in related technologies, including intraoral scanning, digital design software, and three-dimensional (3D) printing.^1^ Traditionally, aligners have been fabricated using thermoforming techniques, while 3D printing was mainly limited to the production of dental models. In this conventional workflow, a thermoplastic material is heated until it becomes pliable and is then formed over a dental model. After cooling and solidification, the material retains its shape, resulting in a customized orthodontic appliance.^2^^,^^3^ This conventional thermoforming has been shown to compromise the mechanical properties of aligner materials.^4^^,^^5^ Consequently, 3D printing has recently emerged as an alternative approach,^6^, ^7^, ^8^ eliminating cumulative errors associated with thermoforming, such as material thinning and mechanical weakening.^4^ In addition, 3D printing enables greater accuracy and precise control over aligner design and thickness, which may enhance treatment efficacy and reduce the need for refinement. Although it requires considerable initial investment, this technology can improve manufacturing efficiency by streamlining workflows, reducing material waste, and simplifying supply chain logistics.^9^^,^^10^ These advancements highlight the need to evaluate the efficacy and precision of 3D-printed materials in delivering orthodontic forces.^11^^,^^12^

The biomechanical behaviour of aligners is controlled by numerous factors, such as thickness,^13^^,^^14^ extension and design of the trimming line,^15^ magnitude and direction of movement.^16^^,^^17^ However, the material type remains the most essential variable associated with the mechanical and clinical efficiency of aligners.^18^, ^19^, ^20^ Conventional thermoformed aligners are made of different types of polymers, such as polyethylene terephthalate glycol or thermoformed polyurethane.^18^^,^^21^^,^^22^ However, recently, shape memory polymers (SMPs) have been introduced innovatively to overcome the limited aligner step size of conventional aligners and to improve the adaptation.^23^^,^^24^ The new concept involves utilizing the shape recovery of SMPs to apply sustained force to teeth over a wide range of orthodontic movements.^23^^,^^24^

In the standard aligner treatment protocol, patients are instructed to wear their aligner splints continuously throughout the day, removing them solely for meals and oral hygiene routines. Each aligner’s splint is generally worn for 7 to 14 days^25^ before being substituted by the subsequent one.^26^ Consequently, over these 14 days, each aligner is exposed to intermittent mechanical and thermal stresses. Thermal stresses arise from temperature fluctuations within the oral environment. Short-term mechanical stresses occur during aligner insertion and removal, whereas long-term mechanical stresses result from continuous contact between the aligner and malaligned teeth, as well as from occlusal forces generated during tooth contact.^27^

Despite the significant shift in clinical practice following the introduction of 3D-printed aligners, a critical need remains for systematic investigations, particularly in clinically relevant areas. To address this gap, our research team is conducting a comprehensive project evaluating 3D-printed aligners from multiple perspectives, including their mechanical properties, precision, and force transmission. The primary aim of this experimental study is to quantify the normal contact and resultant forces exerted by 3D-printed aligners on Tooth 11 before and after thermomechanical ageing, compared with conventional thermoformed aligners. Additionally, aligner thickness was assessed to evaluate the effect of manufacturing method on thickness accuracy.

## Materials and methods

### Aligner fabrication

**Fig. 1:**
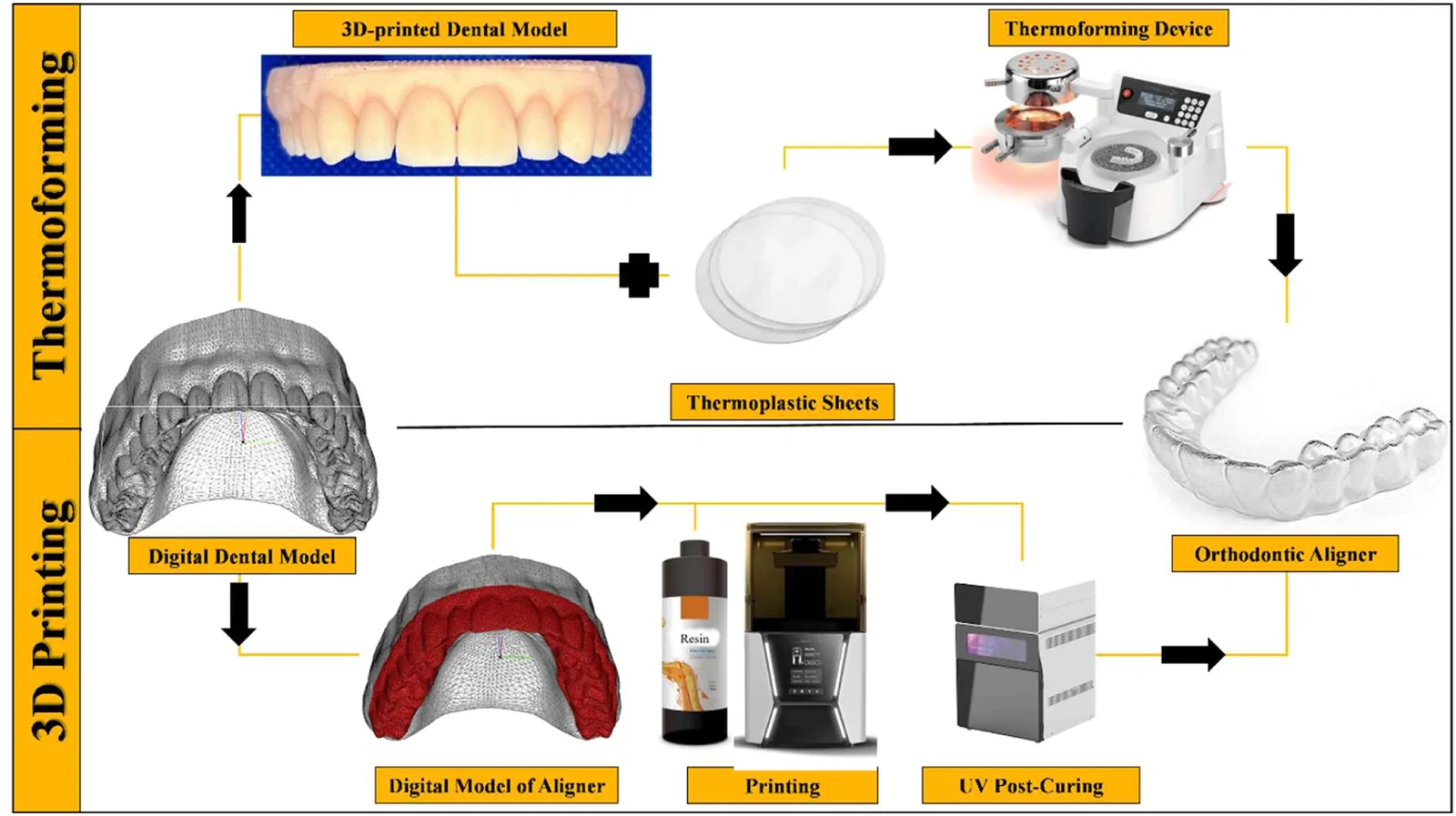
Schematic diagram illustrating the workflow followed in the current study to produce aligners using either thermoforming (Zendura FLX foils) or 3D printing (Tera Harz TC-85 resin).

Two different types of aligner materials were investigated in the current study: one 3D-printed (Tera Harz TC-85; Graphy) and one thermoformed as a control (Zendura FLX [ZF]; Bay Materials). Ten full anatomical aligners (*n* = 10) were made from each material, following each manufacturer’s guidelines as presented in Table 1. The trimline design of all aligners was a straight 2-mm extended design^15^ (Figure 1).

**Table 1:** Thickness, fabrication conditions, and composition of the investigated aligner materials.

Name/thickness	Thermoforming/3D printing conditions	Material composition
Tera Harz TC-85* (TC-85) Thickness: 0.75 mm	3D printing was performed using a DLP-type 3D printer (Uniz NBEE; Uniz) with a layer thickness of 100 µm at an angle of 45° and offset of 0.1 mm. Following the printing process, excess resin was removed by centrifugation using a centrifuge (FO 415-0003; Graphy) for 6 min. Subsequently, postcuring was carried out under nitrogen atmosphere using UV light at a wavelength of 405 nm for 25 min in a dedicated postcuring unit (Tera Harz Cure; Graphy).^23^	Urethane acrylate oligomer with acrylic monomers.
Zendura FLX (ZF) Thickness: 0.76 mm	Thermoforming using a deep-drawing device (Ministar S; Scheu-dental GmbH), Code (162) | Heating for 50 s at 220°C, and pressure-forming at 5.8 bar, then cooling for 60 s.^12^	A three-layer sheet of a soft thermoplastic elastomeric layer between two hard layers of a copolyester (polyurethane-based material, PU).

⁎ Tera Harz TC-85 is certified by CE, KFDA, and FDA.

As presented in Figure 1, the production process of anatomical aligners involves the utilization of a digital model of a maxillary arch, created from a 3D data set (Digimation). This model was exported as an STL file and processed in two distinct ways. First, it was printed using P pro resin (Straumann AG) by a digital light processing 3D printer (P20+; Straumann AG), in 15 mm height from the occlusal table to the base,^2^ for subsequent thermoforming of ZF sheets as described in Table 1. Second, the digital model was used to design full anatomical aligners, utilizing 3D image processing and editing software (3-matic 16.0; Materialise), which were directly 3D-printed from TC-85, as specified in Table 1.

### Force measurements

**Fig. 2:**
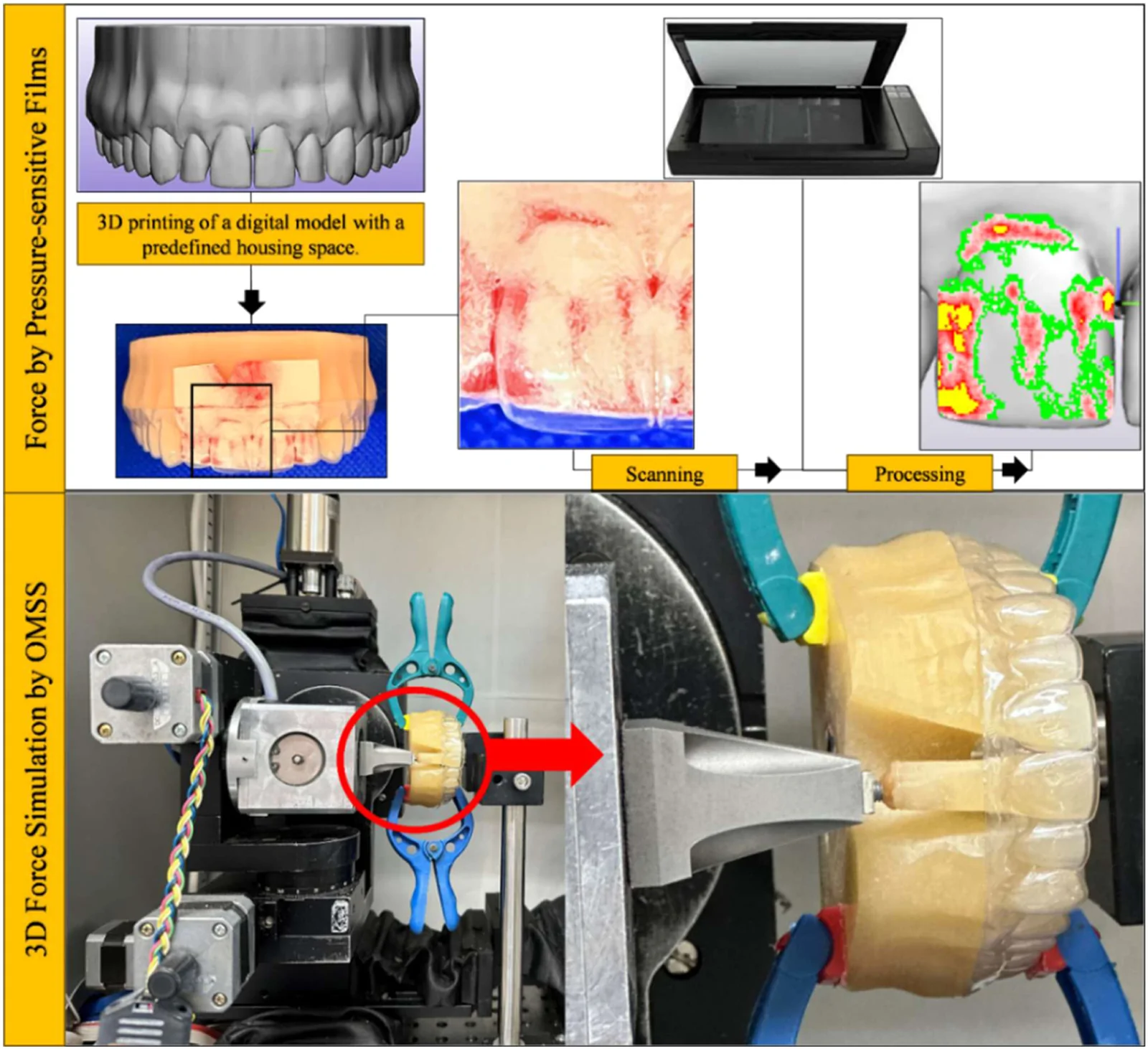
Visual representation of the process for measuring force on the crown of an upper right central incisor (Tooth 11) using Fuji pressure-sensitive film to assess contact normal forces and the orthodontic measurement and simulation system (OMSS) to measure resultant 3D forces.

Force measurements were performed using two complementary techniques, as illustrated in Figure 2. First, low-pressure, pressure-sensitive films (Fuji Prescale Film; Fuji Film) were used to record local contact forces on the coronal facial surface of Tooth 11, according to the protocol detailed in a recent study.^15^ Second, a custom-made biomechanical testing system, the Orthodontic Measurement and Simulation System (OMSS; Oral Technology, University Hospital Bonn), was employed to simulate 3D tooth movements and measure the resultant 3D forces acting on the tooth. The OMSS is designed to quantify forces and moments along all three spatial axes using sensors mounted on motorized stages with full 3D mobility. The OMSS system operates within a temperature-controlled chamber and is computer-controlled, allowing both absolute force measurements and dynamic simulations of orthodontic tooth movement.^28^ The present experiments were conducted under dry conditions at room temperature (≈25°C). Force measurements for each aligner were performed using both testing methods at four predefined time points: before ageing (Day 0) and after thermomechanical ageing on Days 2, 7, and 30.

To conduct mechanical ageing of the aligners, a second malaligned model was created, in which the upper right central incisor (Tooth 11) was bodily translated facially by 0.2 mm (M-model). A total of 20 models of this malaligned version were printed. Thermomechanical ageing was conducted by seating each aligner on the M-models and immersing them in a temperature-controlled circulating water bath (DC5-Haake K15; Thermo-Haake) containing distilled water maintained at 37°C. This protocol ensured standardized and reproducible ageing conditions throughout the experimental period.

For the measurement of normal contact forces using pressure-sensitive films, an additional malaligned resin model was fabricated incorporating a 100 µm clearance around the four incisors and their corresponding gingival regions (housing space) (Figure 2). This housing space was designed to eliminate the influence of the inherent thickness of the pressure-sensitive films on force measurements.^15^ The pressure-sensitive films were carefully positioned within the designated housing space, after which the aligner was gently seated to avoid the application of unintended pressure. Each pressurized film was subsequently scanned using a dedicated scanner (EPSON Perfection V300 series; Seiko Epson) (Figure 2). Digital image processing and force quantification were performed using specialized software (FPD-8010E analysis system; Fuji Film), which enabled direct computation of force distribution across the tooth surface. Normal contact forces (N) were recorded for the incisal (I), middle (M), and cervical (C) thirds of the facial surface of Tooth 11, as well as for the entire facial surface (Figure 2).

To measure the resultant force generated during facial translation of Tooth 11 using the OMSS, an additional duplicate resin model (Technovit 4004; Kulzer) was fabricated. In this model, Tooth 11 was rendered freely movable without mechanical resistance and connected directly to the measuring unit, while the remaining resin model was rigidly fixed (Figure 2). Before each measurement, the tooth was positioned in its neutral, unloaded state and aligned with the sensor axis to ensure the absence of preloading forces. During testing, Tooth 11 was translated in the facio-lingual direction by ±0.2 mm at a constant speed of 0.01 mm/s.

### Aligner thickness measurements

**Fig. 3:**
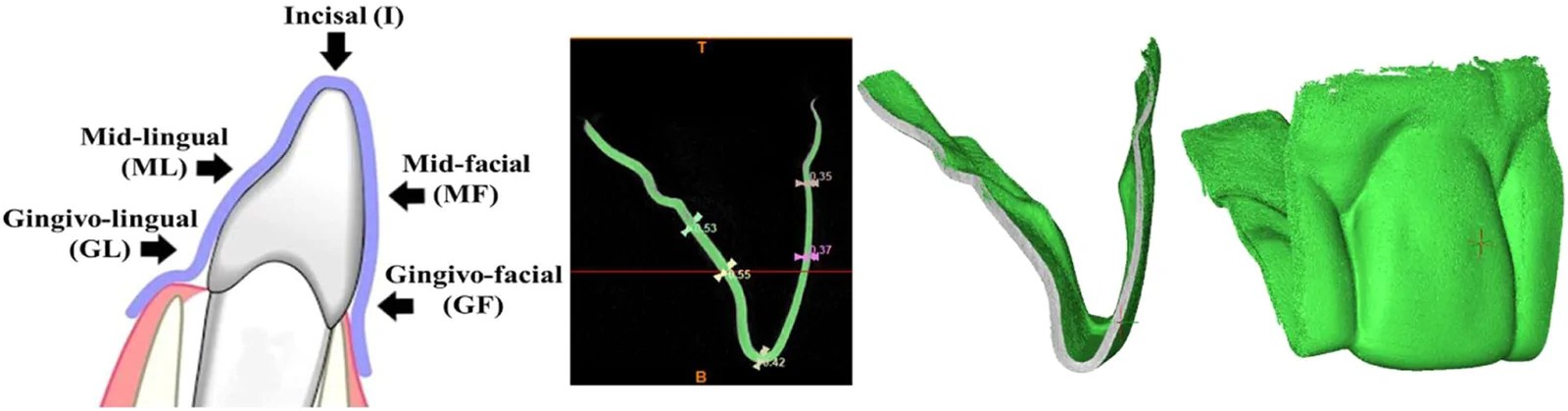
A µCT-processed 3D image for a part of the aligner over Tooth 11 for measurement of aligner thickness by MIMICS software. Thickness measurements were taken at gingivo-facial (GF), midfacial (MF), incisal (I), midlingual (ML), and gingivo-lingual (GL) points.

Aligner thickness over Tooth 11 was also assessed using two complementary methods. First, thickness was measured using a digital caliper (Dial Caliper D; Aura-Dental). Second, microcomputed tomography (µCT) analysis was performed using a high-resolution scanner (Skyscan 1174; Bruker microCT). Scanning was conducted without filters at a voxel resolution of 22 µm, with an accelerating voltage of 50 kVp, a beam current of 800 µA, an exposure time of 22 seconds, and a full 360° rotation with a 0.4° angular step. Each scan comprised approximately 1018 slices and required about 90 minutes to complete. The reconstructed µCT data sets were exported as Digital Imaging and Communications in Medicine files and processed using dedicated 3D medical image analysis software (Mimics 25.0; Materialise). Image visualization and segmentation were performed by radio-opacity thresholding, and a 3D model of each aligner was generated by propagating the selected threshold throughout the image stack. Accurate determination of the radio-opacity threshold was critical for reliable 3D reconstruction and thickness analysis. Thickness measurements were obtained at standardized reference points along the midline of the aligner segment covering the crown of Tooth 11, including the gingivo-facial, midfacial, incisal (I), midlingual, and gingivo-lingual regions (Figure 3).^2^ A single, well-trained operator performed all measurements under strictly standardized and carefully controlled conditions to ensure consistency. Three new full-anatomical aligners were fabricated from each material for thickness assessment (Table 2).

**Table 2:** Experimental design of the study, including the investigated materials, digital and physical models, measurement techniques, and sample sizes.

Category	Description
Materials	• 3D-printed aligners: Graphy (Tera Harz TC-85)
	• Thermoformed aligners (control): Zendura FLX
Digital model	• Maxillary arch STL data set used for aligner design and fabrication
Experimental models	• Ideal resin model (baseline)
	• Malaligned model (Tooth 11 displaced 0.2 mm facially)
	• Pressure-sensitive film model (100 µm housing space)
	• OMSS model (Tooth 11 freely movable)
Measurement methods	• Pressure-sensitive films: normal contact forces (facial surface of Tooth 11)
	• OMSS: resultant 3D forces during ±0.2 mm facio-lingual movement
	• Thickness: digital caliper and µCT
Sample size	• Force measurements: *n* = 10 aligners per material
	• Thickness measurements: *n* = 3 aligners per material

### Statistical analysis

Sample size was calculated using the software G × Power (version 3.1), with a study power of 80% and a significance level of 0.05. The effect size (*n* = 5) was derived based on a study by Cervinara et al.^29^ The Shapiro–Wilk test was used to check normality. Data exhibited parametric distribution and were presented in mean and standard deviation. Statistical analysis was carried out using IBM SPSS Statistics (IBM). Analysis was conducted using one-way ANOVA followed by posthoc Tukey HSD test. The significance level (*α*) was *P <* .05.

## Results

**Fig. 4:**
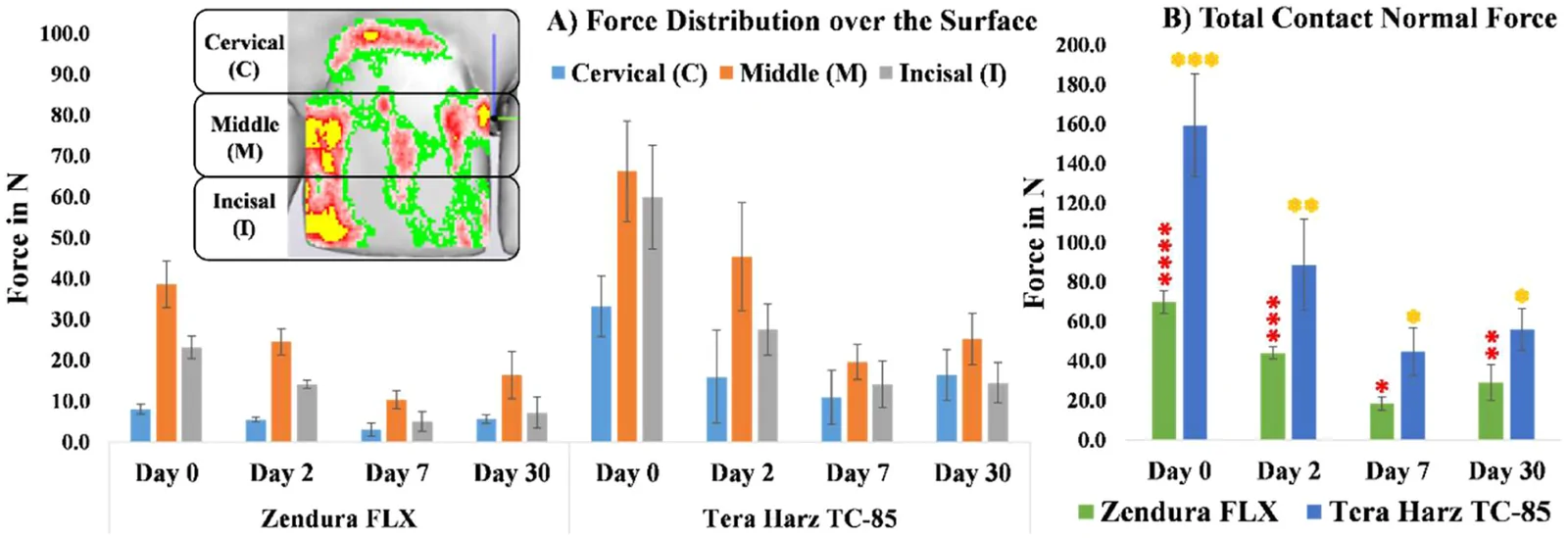
(A) Distribution of contact normal forces across the cervical (C), middle (M), and incisal (I) regions of the maxillary right central incisor for Zendura FLX and Tera Harz TC-85 aligners after thermomechanical ageing. (B) Total contact normal forces measured for both aligner materials at each evaluation time point. Values are presented as mean ± SD. Asterisks indicate statistically significant differences (*P* < .05) (yellow colour for within Tera Harz groups and red colour for within Zendura FLX groups).

In Figure 4, analysis of stress distribution revealed a nonuniform pattern across the facial surface of Tooth 11. For both materials, significant differences were observed among the cervical (C), middle (M), and incisal (I) regions, with stress values consistently and significantly lowest at the cervical third. Moreover, TC-85 aligners exhibit a considerably higher transmission of normal contact force, as measured by pressure-sensitive films, compared to ZF aligners throughout all measurement periods. Before ageing (Day 0), the normal contact force, transmitted onto the whole coronal facial surface of Tooth 11, was 69.8 N for ZF and 149.7 N for TC-85. Over the course of 30 days of thermomechanical ageing, both materials exhibited a significant decay in force from the second day, decreasing substantially till the 30th day. Both materials showed uneven distribution of contact forces, with higher concentrations of force in the middle and incisal one-third compared to the cervical one-third. The force difference between the surface thirds (incisal-cervical or middle-cervical) was notably lower with TC-85 than with ZF. However, this force discrepancy between the three thirds within each material diminished over time, particularly on Days 7 and 30.

**Fig. 5:**
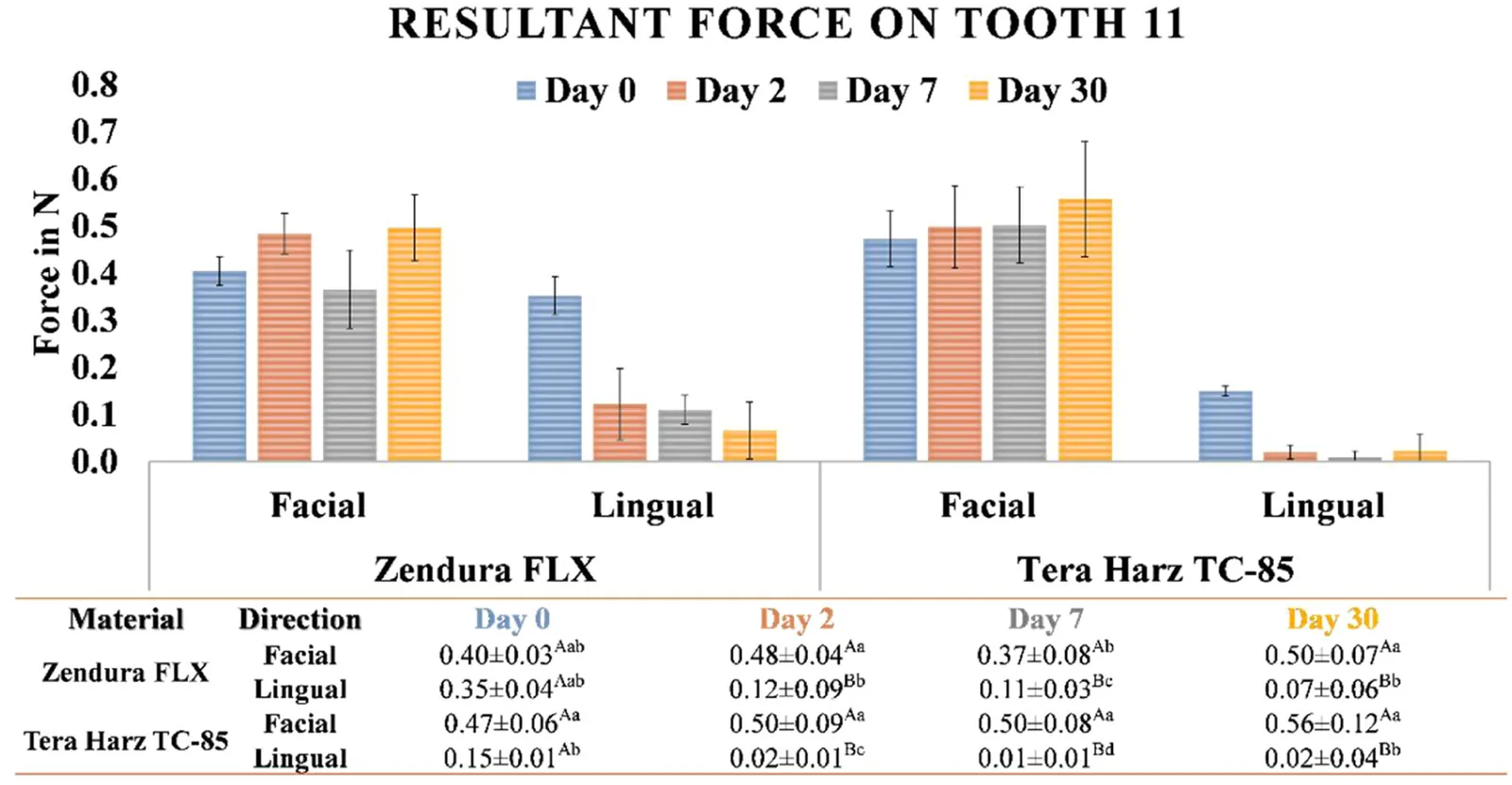
Resultant forces transmitted to Tooth 11, when translated in facial and lingual directions, by aligners fabricated from two different materials (thermoformed Zendura FLX and 3D-printed Tera Harz TC-85), measured using the Orthodontic Measurement and Simulation System (OMSS) at different ageing time points. Different uppercase and lowercase superscript letters indicate statistically significant differences within the same horizontal row and vertical column, respectively (*P* < .05).

Figure 5 presents the resultant 3D forces measured by OMSS, which ranged from 0.1 to 0.5 N before ageing. Force generation exhibited direction dependence for both materials, with greater forces recorded during facial translation of Tooth 11 than during lingual translation. At Day 0, forces on facial translation did not differ significantly between the two materials, whereas forces on lingual translation were significantly lower for TC-85. Over 30 days of thermomechanical ageing, forces on facial translation remained stable for both materials, while forces on lingual translation declined significantly from Day 2 onwards, with TC-85 exhibiting greater force decay than ZF.

**Fig. 6:**
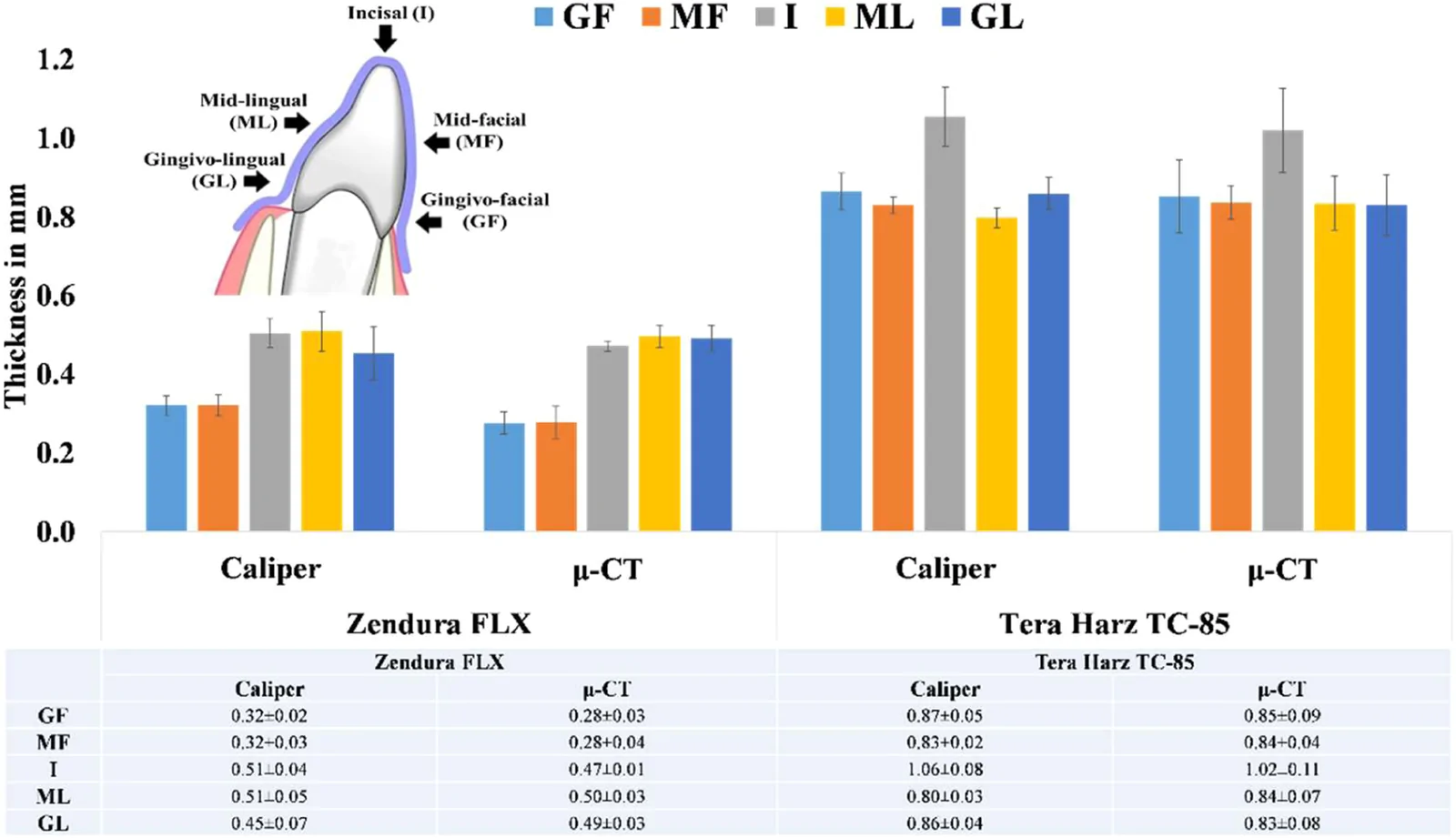
Thickness of aligners fabricated from two different materials (thermoformed Zendura FLX and 3D-printed Tera Harz TC-85) measured at five standardized points along the midline of Tooth 11 using two methods: digital caliper and microcomputed tomography (µCT).

As shown in Figure 6, thickness measurements obtained using a digital caliper and µCT were consistent and mutually validating. The initial thickness of ZF thermoformed sheets was 0.76 mm, whereas the 3D-printed TC-85 aligners were designed at 0.75 mm. Following thermoforming, ZF aligners exhibited substantial thinning, ranging from 0.3 to 0.5 mm (33%-60% reduction), with the least thinning at the incisal edge (0.5 mm; 30%) and the greatest at the facial surface (0.3 mm; 60%). In contrast, TC-85 aligners showed a thickening effect, with thickness increasing to 0.8 to 1.1 mm (6.3%-29.2%), most notably at the incisal edge (29.2%).

## Discussion

Ongoing research into the biomechanical properties of orthodontic aligners aims to refine treatment protocols and optimize clinical outcomes. These studies leverage both numerical and experimental methodologies, including finite element analysis,^17^^,^^30^ alongside experimental setups like pressure-sensitive films^31^ and customized multiaxis force/torque biomechanical systems,^32^^,^^33^ similar to OMSS used in the present study.^28^ A detailed discussion of the methodology applied in the current study has been comprehensively described in our previous publications.^15^^,^^34^ Hence, to avoid redundancy and unnecessary manuscript length, the current discussion focuses primarily on the interpretation of the results.

The limited amount of tooth movement achievable with each conventional thermoformed aligner necessitates a larger number of splints per treatment, thereby increasing overall costs and substantially elevating plastic consumption, with associated environmental concerns. In this context, the integration of SMPs and 3D printing into aligner therapy may provide a more precise, cost-effective, and environmentally sustainable alternative.^23^^,^^24^ Accordingly, the primary aim of the present experimental study was to characterize the force system of a directly 3D-printed aligner (Tera Harz TC-85) in comparison with a thermoformed aligner (ZF), both before and throughout 30 days of thermomechanical ageing, and additionally to report on aligner thickness.

The choice of a 30-day study period is based on the specific SMP properties of the tested 3D-printed aligners. Unlike conventional thermoformed aligners, which are typically worn for 7 to 14 days, SMP-based aligners have the theoretical potential to enable greater incremental tooth movement over an extended wear period without additional aligner changes.^8^^,^^35^ The period of 30 days was therefore chosen to investigate thermomechanical stability and force development under extended clinically relevant conditions. The aim was not to develop a direct clinical recommendation for extended wear but rather to evaluate whether the initial and residual forces could be maintained over a longer period of time. Since the force drop most likely occurs within the first 48 hours, as reported in previous studies^25^^,^^27^^,^^36^ and also observed in the present results, measurements taken at Days 0, 2, 7, and 30 were considered sufficient. Therefore, the inclusion of an additional measurement at Day 14 was deemed unnecessary.

Optimal orthodontic force consists of a light continuous load applied to teeth to achieve efficient movement while minimizing adverse effects on the surrounding tissues.^37^ Force generation by aligners arises from a programmed geometric mismatch between the aligner and the tooth surface, resulting in force transmission over broad and poorly defined contact areas rather than well-defined points of application. The complex 3D morphology of the tooth further exacerbates this effect, leading to heterogeneous contact conditions and nonuniform stress distribution.^29^ As a result, localized areas of intimate contact act as stress concentration zones, while relieved regions contribute minimally to effective force delivery, explaining the uneven stress patterns observed in the current pressure-sensitive film analysis, which comes in agreement with Cervinara et al.^29^ During simulated facial bodily translation of Tooth 11, none of the tested aligner materials produced the force-moment system required for true bodily movement. Specifically, the magnitude and distribution of cervical forces were insufficient to counterbalance the incisal forces relative to the tooth’s centre of resistance, resulting in a net tipping moment.^15^^,^^29^

The normal contact forces measured in the present study were 69.8 N for ZF and 149.7 N for TC-85 before ageing, markedly exceeding the values commonly reported in the literature as optimal orthodontic force (0.1-1.2 N).^37^ This discrepancy is primarily attributable to the use of pressure-sensitive films, which capture highly localized, one-dimensional contact forces at a single tooth surface. In contrast, aligners generate complex 3D force systems distributed across multiple tooth surfaces and vectors, resulting in substantially lower net resultant forces, as measured by OMSS. Nonetheless, this method is valid, since all groups were assessed and compared under identical, standardized experimental conditions.^2^

Although Cervinara et al.^29^ have employed the same measurement technique, their results were reported in terms of pressure (MPa) rather than force. When accounting for the average pressurized contact area in the current experiment (≈40 mm²), the reported values are biomechanically comparable to Cervinara et al.’s findings of approximately 3.3 MPa, because force (N) is the product of pressure (MPa) and contact area (mm²). This conversion allows a meaningful biomechanical comparison between studies despite differences in reporting units. Likewise, a clinical study by Barbagallo et al^38^ reported contact forces of approximately 28.0 N for a thermoformed aligner using pressure-sensitive films. The reported lower value can be attributed to the presence of the periodontal ligament, which acts as a compliant force-damping structure, thereby absorbing and redistributing loads and explaining the discrepancy between in vivo and in vitro measurements.

Optimal aligner adaptation and retention are critical for the transmission of high-precision forces, directly influencing the accuracy and efficiency of orthodontic tooth movement. Beyond the active forces elicited by geometric mismatch, passive forces emerge from the intimate interface between the aligner and the 3D-printed model.^39^ These passive forces serve as a clinical indicator of mechanical engagement and retention.^20^ Notably, TC-85 has been shown to exhibit superior adaptation compared to conventional thermoformed aligners,^40^ a characteristic that likely accounts for the elevated contact forces observed in TC-85 relative to ZF. This is further supported by thickness measurements, which reveal that TC-85 is nearly double the thickness of ZF, thereby facilitating higher force transmission.^17^

Utilizing similar biomechanical systems for measuring forces of TC-85, previous studies have reported varying force magnitudes. Grant et al^41^ recorded approximately 1.4 N at a 0.5 mm faciolingual displacement, while Lee et al^42^ and Hertan et al^43^ measured 0.99 N (at 0.25 mm) and 1.2 N (at 0.2 mm), respectively. Additionally, Sharif et al^12^ observed forces ranging from 0.4 to 1.7 N at a 0.4 mm facial translation. In the present study, a 0.2 mm deflection of Tooth 11 generated preageing forces between 0.1 and 0.5 N for both materials. These results align with the previous studies and fall within the recommended orthodontic force range, confirming that both TC-85 and ZF generate clinically acceptable force levels. The discrepancies observed across studies likely stem from variations in experimental design, the specific biomechanical systems employed, and the magnitude of programmed tooth deflection.

Understanding material behaviour under multidirectional tooth movements is essential, as force generation by orthodontic aligners is direction-dependent. This behaviour is primarily influenced by tooth morphology and the resulting aligner–tooth contact geometry, which produces different load transmission patterns in different directions.^17^ This may also explain the need for movement-specific overcorrection strategies.^44^ OMSS measurements in this study demonstrated that TC-85 delivers substantially lower lingual forces than ZF. This disparity is likely a function of differing material properties, specifically, the elastic modulus and thermomechanical behaviour.^45^ Because ZF possesses a higher flexural modulus and a higher glass transition temperature (Tg) than TC-85,^23^ it behaves as a more rigid body. This rigidity causes ZF to concentrate stress at areas of maximum tooth prominence (such as lingual cingulum), whereas the greater flexibility of TC-85 allows for broader stress distribution, resulting in a lower measured resultant force in the lingual direction.

In the present study, both thermal and mechanical loading were employed, as the sole application of thermal ageing has minimal impact on force generation by orthodontic aligners, but the cumulative effects of repeated mechanical loading play a significant role in force decay.^27^ Water absorption facilitates molecular diffusion between polymer chains, increasing their mobility, a process further accelerated by elevated temperatures. Besides, under continuous mechanical loading, the formation of microcracks and material creep enhances water penetration, synergistically weakening the polymer framework. This degradation manifests significant force decay and accelerated stress relaxation, with the extent of these changes largely dictated by the polymer’s composition and Tg.^27^^,^^36^

Interestingly, while ageing exerted a significant impact on normal contact forces, its effect on resultant forces was different. This divergence is explained by the distinct biomechanical mechanisms underlying each measurement. Pressure-sensitive sheets quantify localized normal contact pressure at the aligner–tooth interface. Normal contact force is primarily a function of aligner adaptation and thickness,^20^ which may decrease after ageing because of polymer relaxation, reduced adaptation, and loss of localized elastic energy.^36^ In contrast, the OMSS measures the net 3D resultant force transmitted to the tooth. Therefore, localized reductions in contact pressure do not necessarily result in a proportional decrease in the resultant force. In addition, strain-induced hardening or cold working at the highly deformed aligner segments may partially preserve the facial force component despite the reduction in local contact pressure.^27^^,^^36^^,^^46^ However, the ageing effect remained significant in the lingual direction, particularly for TC-85. This is likely influenced by the Tg; while ZF possesses a Tg of approximately 107°C,^23^ well above the 37°C ageing environment, the Tg of TC-85 is remarkably lower at 42.5°C,^23^ making it more susceptible to force decay. Although this may influence the clinical performance of the aligner, the reported shape-memory properties of TC-85 at intraoral temperatures may partially mitigate this effect by promoting shape recovery and reestablishing adaptation during extended wear.^12^

Aligner thickness critically determines stiffness, force magnitude, and the predictability of tooth movement.^20^ Also, thickness variations resulting from thermoforming vs direct 3D printing produce biomechanically relevant differences that directly affect force delivery.^20^ Current results showed that in conventional thermoformed aligners, such as ZF, deep drawing of thermoplastic sheets over the dental model results in pronounced and nonuniform thinning—particularly in the middle and gingival regions (up to 60%-75%), with comparatively less thinning at the incisal edge (≈20%), in agreement with previous reports.^2^^,^^7^^,^^15^ This uneven reduction in thickness decreases aligner stiffness, increases flexibility and deformation, and may compromise force delivery, as observed in both pressure-film and OMSS measurements, thereby reducing treatment predictability. In contrast, the 3D-printed aligners demonstrated a more homogeneous thickness distribution. The uneven stress patterns, observed in pressure-sensitive film analysis, are therefore more plausibly attributed to the complex 3D topography of the tooth surface rather than to thickness variability.^15^ Nevertheless, the combination of homogeneous thickness and improved adaptation to the dental model.^40^ enhances the biomechanical behaviour of 3D-printed aligners, allowing more controlled force transmission and improved regulation of tooth movement.^20^

Current measurements revealed a thickness increase in TC-85 aligners to 0.8 to 1.1 mm (6.3%-29.2%), most pronounced at the incisal edge. Consistently, Lee et al^24^ reported up to a 54% thickness reduction in thermoformed PET-G aligners and an approximately 12% increase relative to the planned thickness in 3D-printed TC-85 aligners. This thickening in printed aligners is likely related to resin overbuilding and polymerization effects inherent to additive manufacturing, including light scattering and postcuring-induced densification.^47^ Given the nonlinear dependence of aligner stiffness on thickness,^48^ even minor overbuilding can disproportionately increase flexural rigidity, affecting force magnitude. The increased incisal thickness may therefore enhance local stiffness, potentially improving force transmission during intrusion and acting as an integrated bite plane, which may be advantageous for posterior intrusion mechanics. Conversely, potential drawbacks include reduced patient comfort, greater difficulty in aligner insertion and removal, and the risk of localized stress concentrations, which could compromise fit or induce unintended tooth movements.

The present study has several limitations. Most notably, force measurements were conducted at room temperature and in dry conditions, thereby excluding the shape-memory effect of TC-85 reported in previous studies^12^^,^^23^ as well as the effect of moisture,^25^ which can substantially alter the material’s biomechanical behaviour. Nonetheless, adapting the current experimental methodology to controlled elevated temperatures and wet conditions was technically challenging. Another limitation is the inherent high sensitivity of the pressure-sensitive film technique, as previously reported,^15^ which may influence measurement reliability. Furthermore, the absence of periodontal ligament and bone simulation likely resulted in overestimated force values, particularly in pressure-sensitive films. Moreover, despite standardized fabrication protocols, inherent geometric inaccuracies associated with both thermoforming and 3D printing cannot be fully excluded. Furthermore, the biomechanical evaluation was restricted to Tooth 11. Although this standardized approach enabled accurate and reproducible measurements, it does not fully represent the complex force distribution within a complete dental arch. Future studies using multitooth or full-arch models are warranted to confirm the clinical applicability of these findings.

Another limitation of the present study is that the interfacial gap between the aligner and the tooth was not quantified. Since contact force transmission is influenced by aligner fit, which may vary between thermoformed and 3D-printed aligners because of their different manufacturing processes, part of the observed force differences may be attributable to fit accuracy rather than material properties alone. Future studies should combine force measurements with quantitative assessment of aligner fit using techniques such as micro-CT or optical scanning. Finally, the in vitro conditions could not fully replicate the complex oral environment. Factors such as saliva, pH fluctuations, enzymatic activity, masticatory loading, and intraoral temperature may influence the mechanical behaviour and force delivery of aligners in vivo.

Future research should focus on optimizing 3D-printing parameters to mitigate unintended wall thickening, alongside integrating AI tools to customize aligner thickness for specific tooth movements. Additionally, investigating force systems and ageing behaviour under clinically relevant temperatures is essential. To deepen the understanding of aligner biomechanics, further studies should quantify contact forces across various dental surfaces, particularly the lingual aspect. Finally, large-scale longitudinal trials are needed to clarify how thermomechanical ageing affects the chemical, physical, and mechanical properties of these materials.

## Conclusion

Within the limitations of the present experimental study, the following conclusions can be drawn:

1. 3D-printed TC-85 aligners exhibited higher initial contact forces compared to thermoformed ZF aligners. However, these differences cannot be attributed to the elastic modulus alone, as aligner force delivery is influenced by the combined effects of material properties, postmanufacturing thickness, and aligner adaptation.
2. Thermomechanical ageing induced significant force decay in both materials, with the most pronounced reductions observed in lingual resultant forces.
3. Manufacturing-related thickness variations differed markedly between the two aligner systems. Thermoforming produced significant material thinning, whereas 3D printing resulted in localized thickening, which may have contributed to the observed differences in force generation.

## Author contributions

*Conceptualization, methodology, writing – review and editing, writing – original draft, investigation, validation, data curation*: Elshazly. *Writing – review and editing, supervision, resources*: Alhotan, Kim. *Writing – review and editing, data curation, resources*: Shalabi. *Writing – review and editing, data curation*: Elshennawy, Diraz. *Writing – review and editing, data curation, supervision, resources*: Puchert, Kim. *Writing – review and editing, data curation, supervision, visualization, validation, resources*: Şen. *Read and agreed to the published version of the manuscript*: All authors.

## Ethics statement

This article does not contain any studies on human or animal subjects.

## Funding

Ongoing Research Funding Programme (ORF-2026-790), King Saud University, Riyadh, Saudi Arabia.

## Data availability

The data that support the findings of this study are openly available in Mendeley Data at http://doi.org/10.17632/zc5xj6pgy2.1.

## Declaration of generative AI and AI-assisted technologies in the writing process

During the preparation of this work, the authors used ChatGPT and the Gemini AI tools to improve the readability of the English language. After using this tool, the authors reviewed and edited the content as needed and take full responsibility for the content of the publication.

## Conflict of interest

The authors declare that they have no known competing financial interests or personal relationships that could have appeared to influence the work reported in this article.
